# Factors associated with uptake of contraceptives among HIV positive women on dolutegravir based anti-retroviral treatment-a cross sectional survey in urban Uganda

**DOI:** 10.1186/s12905-022-01842-7

**Published:** 2022-06-27

**Authors:** Leah Mbabazi, Mariah Sarah Nabaggala, Suzanne Kiwanuka, Juliet Kiguli, Eva Laker, Arthur Kiconco, Stephen Okoboi, Mohammed Lamorde, Barbara Castelnuovo

**Affiliations:** 1 Infectious Diseases Institute, P.O. Box 22418, Kampala, Uganda; 2 Makerere University College of Health Sciences, P.O. Box 7072, Kampala, Uganda

**Keywords:** Dolutegravir, Anti-retroviral treatment, Contraceptives uptake

## Abstract

**Background:**

In May 2018, following the preliminary results of a study in Botswana that reported congenital anomalies in babies born to HIV-positive women taking dolutegravir drug, the WHO issued a teratogenicity alert. However, there are scarce data on the impact of this guidance on contraceptive uptake among women taking dolutegravir. We assessed the uptake of contraceptives in HIV-positive women of reproductive age on dolutegravir regimens.

**Methods:**

We conducted a cross-sectional survey from April 2019 to July 2019 in five government health facilities in central Uganda, where dolutegravir-based regimens were offered as the preferred first-line antiretroviral treatment. We randomly selected 359 non-pregnant women aged 15–49 years taking dolutegravir-based regimens and interviewed them using semi-structured interviewer-administered questionnaires. We collected data on demographics, contraceptive use, individual, social, and health system factors. We described patients’ characteristics using descriptive statistics and assessed factors associated with contraceptive uptake using a modified Poisson regression model.

**Results:**

A total of 359 women were included in the study. The mean age was 37 years (standard deviation = 6.8) and overall contraceptive uptake was 38.4%. The most utilized method was injectable method at 58.4% followed by condoms (15%), intrauterine device (10.7%), pills (6.4%), implants (5.4%), and sterilization (0.7%). Predictors for contraceptive uptake were parity of 3–4 children (Adjusted Prevalence Ratio (APR) = 1.48, 95% confidence interval (CI): 1.14, 1.92) in reference to those with 1–2 children. There was reduced contraceptive uptake in women of the age range 40–49 years (APR = 0.45, CI: 0.21–0.94) compared to those aged 15–24 years. Unemployed women were less likely to use contraceptives (APR: 0.6, CI: 0.42- 0.94) than the formally employed. Contraceptive uptake was lower among women who did not discuss family planning with their partners (APR = 0.39, CI: 0.29–0.52) than those who discussed family planning with their partners and women who did not receive family planning counseling (APR = 0.56, CI: 0.34–0.92) than those who received family planning counselling.

**Conclusion:**

We observed a low-level uptake of contraceptives, with injectables as the most used method. Family planning counseling and partner discussion on family planning were associated with contraceptive uptake among the women who used dolutegravir-based regimens. There is a need for more strategies to integrate FP services and increase male involvement in HIV care programs.

## Background

Globally, HIV affects mostly women and girls as they contribute 53% of the 37.7 million people living with HIV. Sub-Saharan Africa (SSA) accounts for 67% of the people living with HIV, of which women and girls account for 63% of all new HIV infections [1]. In Uganda, HIV disproportionately affects women, with prevalence at 8.8% compared to 4.3% in men, with the highest prevalence in age groups of 35–49 years (12.9%) [2]. Moreover, women in SSA experience unplanned pregnancies which can contribute to new paediatric HIV infections [3]. The public health burden of unintended pregnancies in HIV-positive women is well demonstrated in Uganda; where the prevalence of unplanned pregnancies among women living with HIV was estimated to be 41% [4]. This is a challenge in the elimination of mother-to-child transmission of HIV (eMTCT). Delivery of family planning (FP) services to HIV-positive women remains inadequate because of the parallel nature of FP and HIV services, with an unmet need for contraception of 41.2% compared to 28% of the general population [5].

The eMTCT strategy advocates a four-pronged approach: primary prevention of HIV infection among women of childbearing age; prevention of unintended pregnancies among HIV positive women; reduction of MTCT among HIV positive pregnant women by starting or keeping them on effective antiretroviral treatment (ART), care, support; and treatment for HIV positive women and their families [6]. Integrating FP into HIV care programs has been an approach to make both FP and HIV care services more accessible to HIV-positive women and couples living with HIV [7].

In 2016, the World Health Organisation (WHO) issued new ART guidelines recommending tenofovir/lamivudine/dolutegravir (TDF/3TC/DTG) as a first-line regimen because of its high efficacy and fewer side-effects compared to the previously used efavirenz based regimen [8]. However, a warning was issued by WHO in May 2018, citing a potential risk of neural tube defects in babies born to women who used DTG regimen during pregnancy [9]. This concern stemmed from the preliminary analysis of a birth surveillance study in Botswana, which reported 4 cases of neural tube defects out of 426 infants born to women who were on DTG during pregnancy [10].

Therefore, WHO amended the 2016 ART guidelines recommending that women of childbearing age could receive DTG along with consistent and reliable contraception and those who intended to conceive could receive EFV-based ART [11]. Following this caution, many countries including Uganda slowed down the transition process and took a conservative approach on women’s access to DTG [12]. Subsequent data from Tsepamo study in Botswana demonstrated that the risk for neural tube defects was lower than the original report that triggered the teratogenicity alert [13]. However, there was still a demand for this to be an opportunity to strengthen FP services for HIV programs.

Currently, there is limited information on the uptake, knowledge, and experiences of the women on FP after switching to DTG based regimens. This study, therefore, aimed to assess the contraceptive uptake and its related factors among HIV-positive women who were receiving DTG based regimens in urban Uganda.

## Methods

### Study setting

This was a cross-sectional survey conducted from April to July 2019 at the five ART clinics of the Kampala Capital City Authority (KCCA) Uganda, supported by the Infectious Diseases Institute (IDI).

### Study population

We included HIV-positive women aged 15–49 years receiving DTG based regimens; women who were pregnant were excluded. We estimated the study sample size of 393 using the Kish formula [14]; at 95% Confidence Interval, Z value of 1.96, 0.05 margin of error and proportion of uptake of FP among HIV positive females (P) as 36% [15]. Stratified random sampling with proportional allocation of participants per the five facilities was used to select eligible participants for enrolment into the study.

### Data collection

The eligible participants were invited to the study sites for data collection via telephone calls. We carried out data collection using semi-structured interviewer-administered questionnaires pretested on five participants who were later not included in the study. All data collection documents were translated into the local language (Luganda). The questionnaires were validated using previous cross-sectional studies that assessed contraceptive uptake in HIV-positive women [15]. The questionnaires were crosschecked daily for completeness to ensure quality.

We collected data on the following variables: age in years (stratified as 15–24, 25–29, 30–34, 35–39, 40–44, 45–49), health facility coded as 1, 2, 3, 4 and 5 respectively. Education level (primary, secondary, tertiary, and none), marital status (married, single and cohabiting), employment (employed, unemployed and self-employed) parity as the number of children (1–2, 3–4, 5–6 and None), religion (Anglican, Catholic, Moslems and others category that included Pentecostal, Jehovah witness, Adventists, Buddhists), desire to have children, sexual activity within the last month, awareness of any contraceptive method, awareness of side effects of DTG, discussion of FP with partner and FP counseling at the facility.

We also collected data on contraceptive use and the type of contraceptives used at that time. We defined modern contraception products or medical procedures that are used to prevent the occurrence of pregnancy such as oral contraceptives, the intra-uterine device (IUD), condoms, progestin-only injections, subdermal implant, vaginal barrier methods, spermicides, emergency contraception, female and male sterilization. We defined traditional contraception methods as withdrawal, abstinence, lactation amenorrhea, fertility awareness methods, rhythm method, and moon beads.

### Statistical analysis

For data analysis, we used STATA version 14. We used descriptive statistics to describe participants' characteristics and then modified Poisson regression model to identify predictors of uptake of contraception. Since the prevalence of contraceptive use in this study was common (more than 10%), Poisson model was fit as an alternative to a logistic model. We examined for goodness of fit of the Poisson model, it was modified to a generalised linear model with Poisson family, log link, and we reported robust standard errors for both the unadjusted and adjusted ratios. All independent variables with a p-value < 0.2 at bivariate analysis and those with biological significance were included in multivariable analysis. Prevalence ratios (PR) were calculated at bivariate and multivariable analysis.

## Results

**Fig. 1:**
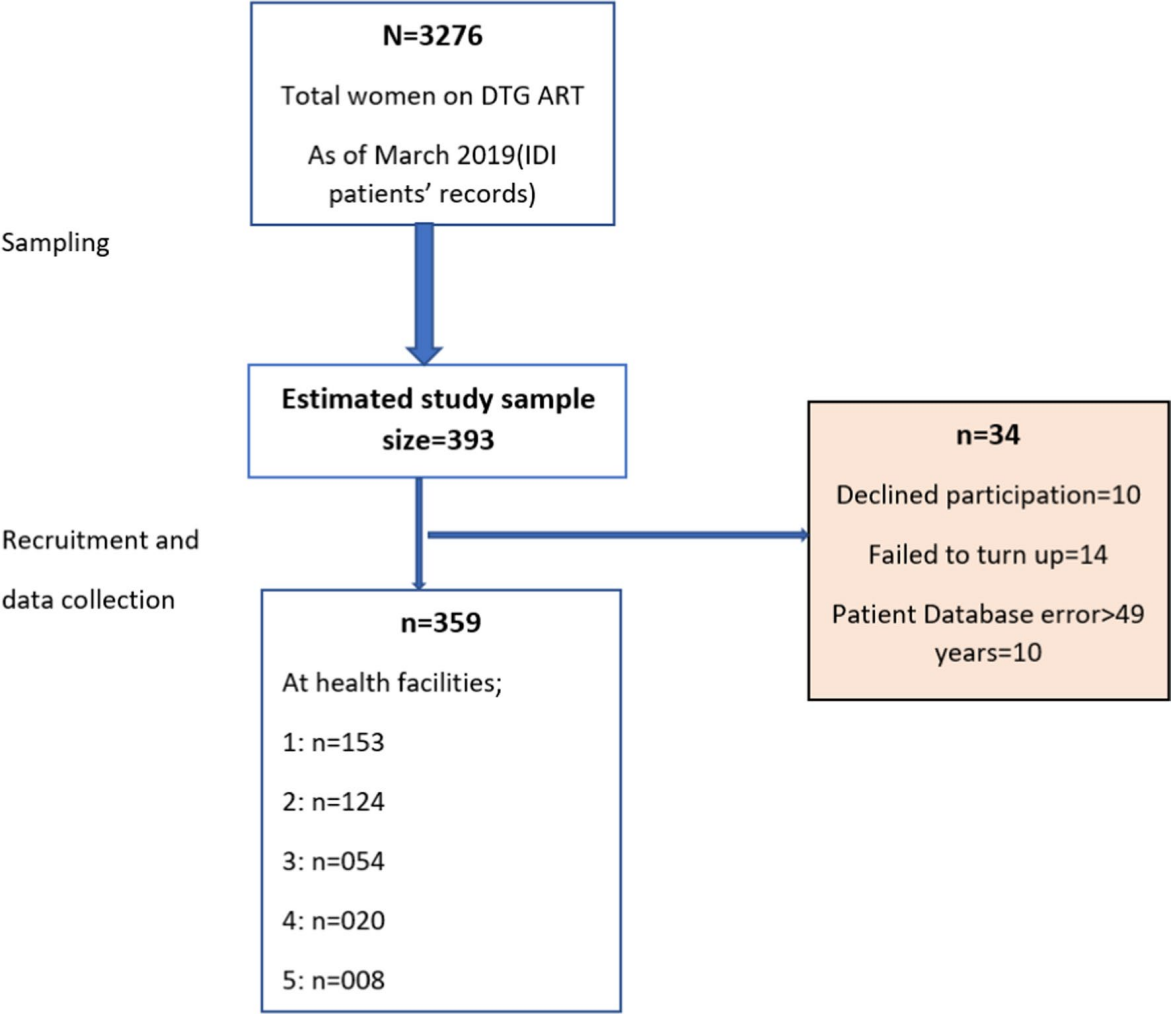
Study flow diagram

Three thousand two hundred and seventy-six women (3276) aged 15–49 years had been started or switched to DTG based ART regimen by March 2019. The sampling procedures are shown using the study flow diagram in Fig. 1.

### Characteristics of respondents and univariate analysis

Participants’ characteristics are described in Table 1. Most respondents (142; 39.6%) belonged to the age category of 40–49 years and the mean age was 37 years (standard deviation (SD):6.8). Half (182, 50.7%) of the respondents had attained a primary level of education while only 18 (5.0%) had attained a tertiary level of education. Most respondents (151, 42.1%) were Anglican and the least (23, 6.4%) were of other religions (Adventists, Buddhists, Pentecostal, Jehovah witness). Most respondents were married (160, 44.6%) children while 25 (7.0%) had no children.

**Table 1:** Characteristics of the respondents and univariate analysis

Variable	Category	Number (column % n = 359)	Contraceptive uptake count (row %)	
			Yes	No
Facility (code)	1	153 (42.6)	63 (41.2)	90 (58.8)
	2	124 (34.5)	32 (25.8)	92 (74.2)
	3	54 (15.0)	30 (55.6)	24 (44.4)
	4	20 (05.6)	7 (35.0)	13 (65.0)
	5	8 (02.2)	6 (75.0)	2 (25.0)
Age	15–24	12 (03.4)	4 (33.3)	8 (66.7)
	25–29	48 (13.4)	30 (62.5)	18 (37.5)
	30–34	64 (17.8)	38 (59.4)	26 (40.6)
	35–39	93 (25.8)	39 (41.9)	54 (58.1)
	40–49	142 (39.6)	27 (19.0)	115 (81.0)
Education level	None	53 (14.8)	12 (22.6)	41 (77.4)
	Primary	182 (50.7)	64 (35.2)	118 (64.8)
	Secondary	124 (34.5)	62 (50.0)	62 (50.0)
Religion	Anglican	151 (42.1)	51 (37.8)	100 (66.2)
	Catholic	123 (34.2)	49 (39.8)	74 (60.2)
	Moslem	62 (17.3)	23 (37.1)	39 (62.9)
	Others	23 (06.4)	15 (65.2)	8 (34.8)
Marital status	Single	81 (22.6)	33 (40.7)	48 (59.3)
	Married	160 (44.6)	78 (48.8)	82 (51.2)
	Divorced/separated	78 (21.7)	23 (29.5)	55 (70.5)
	Widow	40 (11.1)	4 (10.0)	36 (90.0)
Parity (number of children birthed)	1-2	114 (31.7)	48 (42.1)	66 (57.9)
	3-4	149 (41.5)	64 (43.0)	85 (57.0)
	5-6	71 (19.8)	19 (26.8)	52 (73.2)
	None	25 (07.0)	7 (28.0)	18 (72.0)
Employment status	Unemployed	77 (21.5)	55 (49.6)	56 (50.4)
	Formally employed	111 (30.9)	58 (33.9)	113 (66.1)
	Self-employed	171 (47.6)	25 (32.5)	52 (67.5)
Sexual activity (in last one month)	Yes	245 (68.2)	111 (45.3)	134 (54.7)
	No	114 (31.8)	27 (23.7)	87 (76.3)
Discussion of FP with partner	Yes	110 (30.6)	85 (77.3)	25 (22.7)
	No	142 (39.6)	33 (23.2)	109 (76.8)
	N/A (no partner)	107 (29.8)	20 (18.7)	87 (81.3)
Awareness of any contraceptive method	Yes	346 (96.4)	137 (39.6)	209 (60.4)
	No	13 (03.6)	1 (07)	12 (92.3)
Awareness of the side effects of DTG	Yes	272 (75.8)	110 (40.4)	162 (59.6)
	No	87 (24.2)	28 (32.2)	59 (67.8)
Desire to have children	Yes	138 (38.4)	57 (49.1)	59 (50.9)
	No	221 (65.6)	81 (33.3)	162 (66.7)
FP Counselling at the facility	Yes	269 (74.9)	124 (46.1)	145 (53.9)
	No	90 (25.1)	14 (15.6)	76 (84.4)

FP: family planning, DTG: Dolutegravir, Facility codes; Kawaala: 1, Kisenyi: 2, Kiswa: 3, Kitebi: 4, Komamboga: 5

Most respondents (269, 74.9%) reported to have received FP counseling at the facility, 245 (68.2%) reported sexual activity in the last one month and 221 (65.6%) reported they did not want to have any more children. Almost all respondents (343, 96.7%) were aware of at least one contraceptive method and majority of the women 138 (72.6%) were aware of side effects of DTG. Most respondents (142, 39.6%), did not discuss family planning methods with their sexual partners.

### Uptake of contraception

**Fig. 2:**
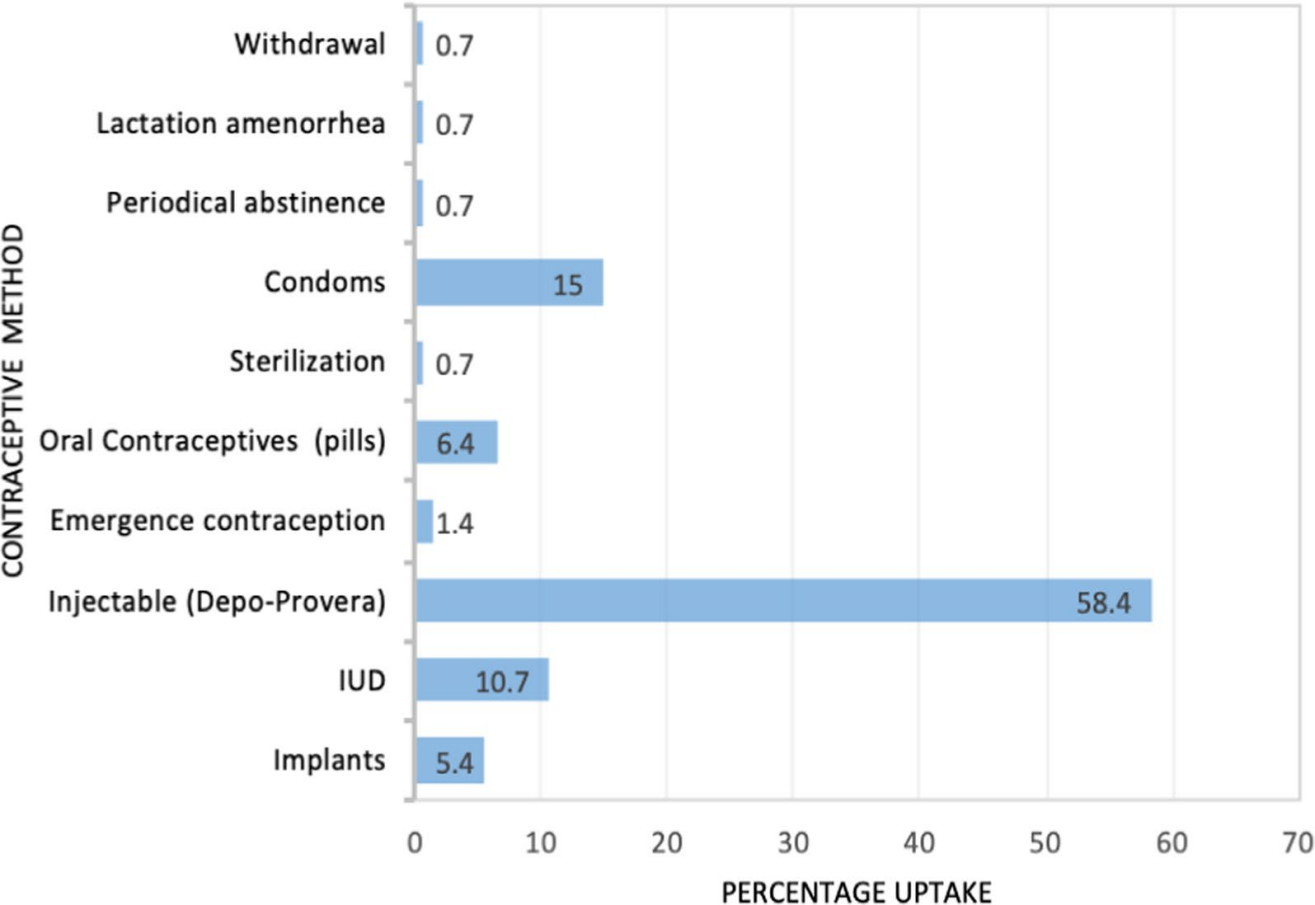
Contraceptive methods used by the women

The majority of women (221/359, 61.6%) were not using any form of contraceptive; only 138/359 (38.4%) indicated use of any contraceptive method. Of the 359, 135 (37.9%) were using modern contraceptives. Figure 2 shows the different FP methods used by the respondents as of the time of the study. The majority (81/138, 58.4%) were using the injectable method. Periodical abstinence, withdrawal, lactation amenorrhea, and sterilization had the least users at 0.7% each.

**Fig. 3:**
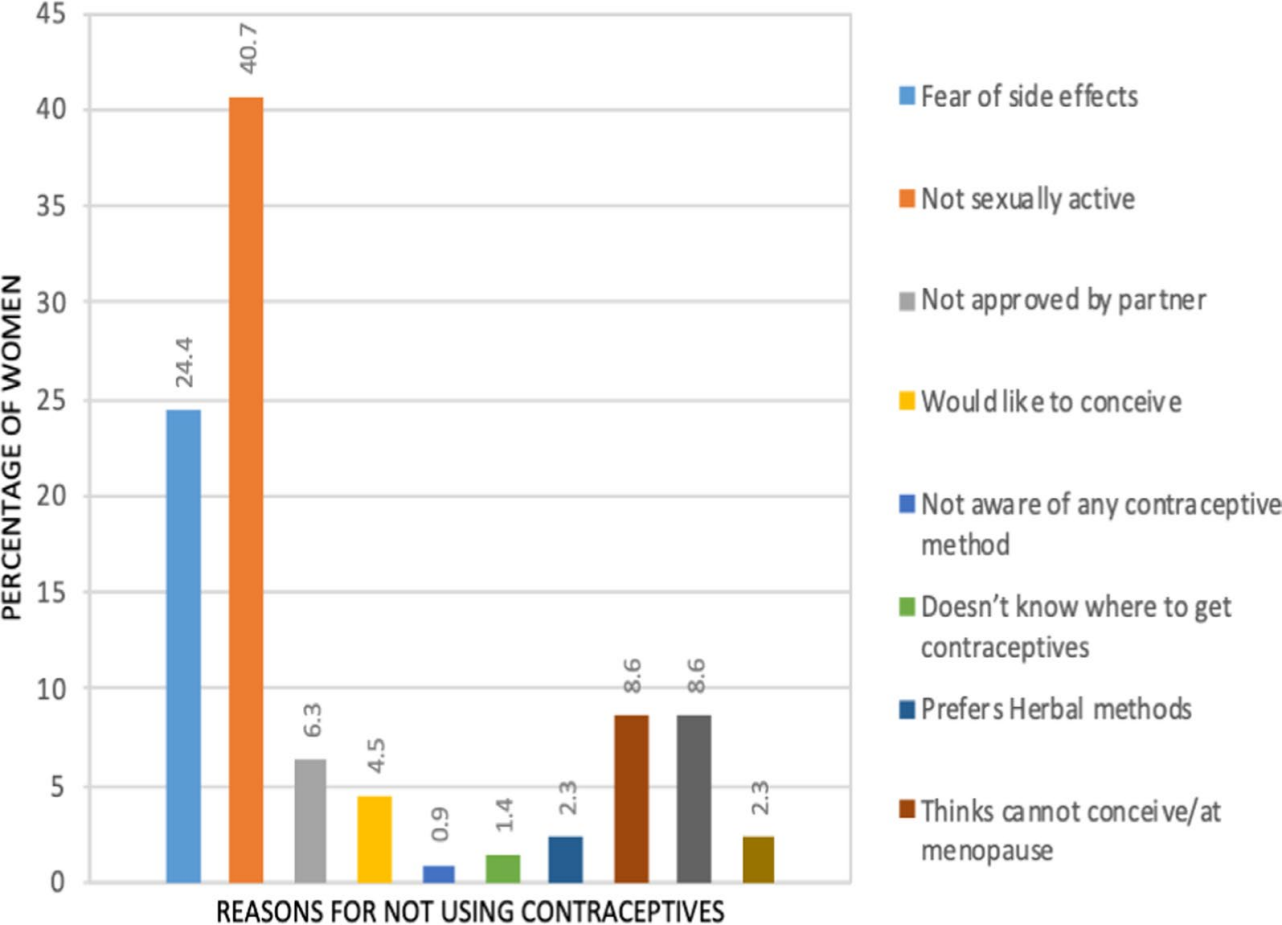
Reasons why women did not use contraceptives

Of 221/359 (61.6%) who did not use contraceptives, 90/221 (40.7%) cited not being sexually active, 54/221 (24.4%) feared side effects, 19/221 (8.6%) anticipated to conceive and 14/22 (6.3%) their partner did not approve FP use. Some participants gave more than one response. Figure 3 shows the different reasons women gave for not using contraceptives.

### Factors associated with uptake of contraceptives

At multivariable analysis, participants who were in the age category of 40–49 years were less likely to use contraception than those of 15–24 years (APR = 0.45, 95% CI: 0.21–0.94), women who had 3–4 children were 1.5 times more likely to use family planning than those who had 1–2 children (APR = 1.48, 95% CI: 1.14–1.92). Unemployed women were less likely to use contraception (APR = 0.63, 95% CI: 0.42–0.94) than the ones who were formally employed. Women who did not discuss FP with their partners were also less likely to use FP (APR = 0.39, 95% CI: 0.29–0.52) than those who discussed FP with their partners. And participants who did not receive FP counseling at the facility were less likely to use FP (APR = 0.56, 95% CI: 0.34–0.92) than those who received FP counselling (Table 2). Health Facility served as a stratification variable thus was included in final model irrespective of its non-statistical significance. The following variables showed no statistical significance; education level, marital status, sexual activity, awareness on contraceptives, desire to have children and awareness on side effects of DTG.

**Table 2:** Factors associated with uptake of contraceptives

Variable	Category	Unadjusted PR (95% CI)	P value	Adjusted PR (95% CI)	P value
Health facility	1	1.00 (ref)		1.00 (ref)	
	2	0.63 (0.44, 0.89)	0.010	0.80 (0.59, 1.10)	0.172
	3	1.35 (0.99, 1.83)	0.054	1.16 (0.84, 1.59)	0.375
	4	0.85 (0.45, 1.59)	0.612	0.87 (0.49, 1.54)	0.622
	5	1.82 (1.17, 2.84)	0.008	0.99 (0.62, 1.56)	0.952
Age	15–24	1.00 (ref)		1.00 (ref)	
	25–29	1.88 (0.82, 4.30)	0.14	0.87 (0.43, 1.75)	0.696
	30–34	1.78 (0.78, 4.07)	0.17	0.81 (0.39, 1.69)	0.570
	35–39	1.26 (0.55, 2.90)	0.59	0.71 (0.36, 1.42)	0.333
	40–49	0.57 (0.24, 1.36)	0.21	0.45 (0.21, 0.94)	0.035
Employment status	Formally employed	1.00 (ref)		1.00 (ref)	
	Self employed	0.68 (0.52, 0.91)	0.008	0.94 (0.73, 1.21)	0.620
	Unemployed	0.66 (0.45, 0.95)	0.026	0.63 (0.42, 0.94)	0.024
Parity	01-Feb	1.00 (ref)		1.00 (ref)	
	03-Apr	1.02 (0.77, 1.36)	0.891	1.48 (1.14, 1.92)	0.003
	05-Jun	0.64 (0.41, 0.99)	0.044	1.33 (0.86, 2.08)	0.201
	None	0.67 (0.34, 1.29)	0.229	1.43 (0.67, 3.05)	0.354
Discuss FP with partner	Yes	1.00 (ref)		1.00 (ref)	
	No	0.28 (0.21, 0.36)	0.000	0.39 (0.29, 0.52)	0.000
FP counselling provided at the facility	Yes	1.00 (ref)		1.00 (ref)	
	No	0.34 (0.20, 0.56)	0.000	0.56 (0.34, 0.92)	0.022

FP: family planning; PR: Prevalence ratio, CI: Confidence interval. Non-significant factors are not included in Table 2

## Discussion

Despite the calls for the need to urgently strengthen reproductive health services after the DTG teratogenicity alert, uptake of contraceptives was still low in our sampled population. The overall level of uptake of contraceptives for this study was 38.4% and uptake of modern contraceptives among the women using contraceptives was 37.9%. Our findings were similar to the results of the 2016 Uganda Demographic Health Survey (UDHS) that reported a contraceptive prevalence rate of 39% [5]. However, findings from other SSA countries, found a higher contraceptive use among HIV-positive women, at 44.3% in Ethiopia [16], 46.4% in Cameroon, [17], and 42.6% in Ghana [18]. The low level of contraceptive uptake in our study could be attributed to the fact that a high proportion of women were reportedly not sexually active and the program of switching to DTG regimens prioritized women aged above 40 years.

Injectable hormonal methods were the most used FP method in this study similar to the general population results of the 2016 UDHS report [5]. This could be explained by the fact that women want short-term reversible methods, due to their reversibility and convenience [16].

Similar to other settings, we found that older participants (40–49 years) were less likely to use contraception than those of 15–24 years, most likely because they were less sexually active and they perceive themselves as less risky of conceiving [19].

Unemployed women were less likely to use contraception than those who were formally employed. Similarly, data from 21 African countries have shown that wealth is positively associated with contraceptive use due to access to private services [20]. Women who had 3–4 children in this study were also more likely to use contraception than the ones with 1–2 children as previously reported.

A high proportion of women did not discuss FP methods with their partners, and they were less likely to use FP in the study. Previous studies demonstrated that partner contribution and approval of FP positively foster contraceptive use among women [21, 22].

In our study, the participants who did not receive FP counseling were less likely to FP use than those who received FP counseling. FP counseling positively affects the attitude to FP use and reduction of myths and misconceptions [23]. When disseminating information on DTG use at HIV caregiving facilities, participants have a chance to express freely and inquire more about reasons for contraceptive use.

This study contributes to the understanding of the uptake of contraceptives among HIV-positive women in Uganda, by identifying the factors affecting contraceptive use in this population. It also contributes to the data on DTG use among women of reproductive age, as we scale up DTG based regimens. Programs should plan and integrate FP counseling and male involvement in reproductive health and HIV care programs.

The major strength of this study is that; it was carried out in five health centres across Kampala. In addition, the study sample of 359 women was adequate to power the study. Therefore, the data obtained from this study is fairly representable and generalizable to the general urban population.

The main limitation of the study was social desirability bias where some participants could have given responses in a favourable manner fearing negative evaluations. The other limitation was that the study did not assess uptake of contraceptives among women who were on other ART regimens and therefore we could not compare uptake of contraceptives among women on DTG versus those on other ART regimens.

In conclusion, contraceptive uptake in this study was low and majority of the women were using injectable methods. The factors that increased the likelihood of contraceptive use were the age of respondents, employment status, parity, discussion of FP with a partner, and FP counseling.

We recommend that HIV programs should promote and offer FP counseling, and where feasible integrate FP services in HIV care. Women should also be encouraged to discuss FP choices with their partners or attend their ART visits with them.

## Data Availability

The datasets generated and analyzed during this study are not publicly available due to the strict data sharing policy of Infectious Diseases Institute. However, they are available from the corresponding author on reasonable request.

## Abbreviations

ART: Anti-Retroviral Therapy
CPR: Contraceptive Prevalence Rate
DTG: Dolutegravir
DMPA: Depot medroxyprogesterone acetate
EFV: Efavirenz
eMTCT: Elimination of Mother to Child Transmission
FP: Family Planning
HC: Health Centre
HIV/AIDS: Human Immunodeficiency Virus / Acquired Immunodeficiency Syndrome
IDI: Infectious Disease Institute
IRB: Institutional Review Board
IUD: Intrauterine Device
KCCA: Kampala Capital City Authority
LMIC: Low and Middle-Income Countries
mCPR: Modern Contraceptive Prevalence Rate
MOH: Ministry of Health
MTCT: Mother to Child Transmission
SD: Standard Deviation
UDHS: Uganda Demographic Health Survey
UNFPA: United Nations Fund for Population Activities
UNICEF: United Nations Children’s Fund
WHO: World Health Organization
